# The Hypolipidemic Effect of *Dalbergia odorifera* T. C. Chen Leaf Extract on Hyperlipidemic Rats and Its Mechanism Investigation Based on Network Pharmacology

**DOI:** 10.1155/2021/3155266

**Published:** 2021-12-27

**Authors:** Hui Mei, Huiming Hu, Yanni Lv, Guangqiang Ma, Fangrui Tang, Zhou Hong, Feng Shao

**Affiliations:** ^1^Key Laboratory of Modern Preparation of Traditional Chinese Medicine, Ministry of Education, Jiangxi University of Chinese Medicine, Nanchang 330004, China; ^2^School of Pharmacy, Nanchang Medical College, Nanchang 330004, China; ^3^Department of Pharmacy, The First Affiliated Hospital of Nanchang University, Nanchang 330006, China; ^4^College of Basic Medical, Jiangxi University of Chinese Medicine, Nanchang 330004, China; ^5^The Research Institute of Tropical Forestry, Chinese Academy of Forestry, Guangzhou 510520, China

## Abstract

**Objective:**

The aim of this study was to explore the hypolipidemic effect and mechanism of *Dalbergia odorifera T. C. Chen* leaf extract.

**Methods:**

The hypolipidemic effect of *D. odorifera* leaf extract was investigated using a hyperlipidemic rat model. Then, its mechanism was predicted using network pharmacology methods and verified using western blotting.

**Results:**

Compared with the levels in the model group, the serum levels of triglyceride (TG), total cholesterol (TC), and low-density lipoprotein cholesterol (LDL-C) decreased significantly, whereas the serum level of high-density lipoprotein cholesterol (HDL-C) increased dramatically after treatment with the extract. The degrees of hepatocyte steatosis and inflammatory infiltration were markedly attenuated *in vivo.* Then, its hyperlipidemic mechanism was predicted using network pharmacology-based analysis. Thirty-five key targets, including sterol regulatory element-binding protein cleavage-activating protein (SCAP), sterol regulatory element-binding protein-2 (SREBP-2), 3-hydroxy-3-methylglutaryl-coenzyme A reductase (HMGCR), low-density lipoprotein receptor (LDLR), and ten signaling pathways, were associated with hyperlipidemia. Finally, it was verified that the extract downregulated the protein levels of SCAP, SREBP-2, and HMGCR, and upregulated protein levels of LDLR.

**Conclusion:**

These findings provided additional evidence of the hypolipidemic effect of *D. odorifera* leaf extract.

## 1. Introduction

According to the World Health Organization, approximately 31% of all deaths are from cardiovascular diseases. These diseases, including atherosclerosis, coronary heart disease, and hypertension, resulting from lipid metabolism disorders [1]. An increase in serum total cholesterol (TC), triglyceride (TG), and low-density lipoprotein cholesterol (LDL-C) levels, and a decrease in high-density lipoprotein cholesterol (HDL-C) levels are the clinical manifestations of lipid metabolism disorders [2]. Currently, the side effects of clinical drugs such as fibrates, statins, and bile acid sequestrants have not been overcome [3]. The advantages of natural products, especially traditional Chinese medicine, include their high efficiency, low toxicity, and easy access. To date, some of them, such as *Crataegus pinnatifida* fruit [4, 5], *Perilla frutescens* leaves [6], and sea buckthorn leaves [7], have been considered attractive materials for treating hyperlipidemia disease.

*Dalbergia odorifera T. C. Chen*, a member of the *Dalbergia* genus, is mainly distributed in the tropics of China. Its heartwood is named “Jiang Xiang” in the Chinese Pharmacopoeia and is used to treat cerebral infarction [8], thrombosis [9], cerebral edema [10], and coronary heart disease [11]. It possesses anti-inflammatory [12], antioxidative [13], antibacterial [14], antitumor [15, 16], and antiplatelet activities [9]; inhibits osteoclast differentiation [17, 18]; and has *α*-glucosidase inhibitory activity [19]. The seed oil from this plant possesses significant DPPH radical scavenging activity, increasing the ferric ion reducing power and ferrous ion chelating ability, and inhibiting linoleic acid peroxidation activity [20]. In addition, its dried leaf is known as a folk medical herb in southern China that improves health [21]. The extract of *D. odorifera* leaves also exhibits antioxidant activity in scavenging DPPH, O_2_^−^, ·OH, H_2_O_2_, and NO_2_^−^ [22]. Up to now, 76 volatile components and 24 nonvolatile components have been isolated from D. odorifera leaves [23–29]. Notably, these components, including biochanin A, genistein, 6-hydroxy-biochanin A, tectorigenin, and prunetin in the latter, possess hypolipidemic activity [30–33]. However, to the best of our knowledge, the hypolipidemic effect of *D. odorifera* leaves has not been investigated in the literature.

Network pharmacology, based on the theory of systems biology and network biology, reveals the complex network relationship between drug targets and diseases using network analysis [34]. It incorporates a series of disciplines and techniques, including genomics, proteomics, and system biology. In recent decades, an analytical method has been widely used to predict the hypolipidemic mechanisms of multiple components, targets, and pathways of medicinal plants. For instance, various compounds from 70% ethanolic extract of *Trigonella foenum-graecum L* seeds mainly work by affecting the activation of the EGFR/AKT/mTOR signaling cascade in the treatment of hyperglycemia and hyperlipidemia [35]. In addition, KEGG analysis indicated that the flavonoid extract of sea buckthorn probably produced hypolipidemic effects by regulating cholesterol metabolism, fat digestion, and absorption, and peroxisome proliferator-activated receptor (PPAR) signaling pathways. The key targets, including PPAR-*α*, PPAR-*γ*, ATP-binding cassette transporter A1, carnitine palmitoyl-transferase 1A, sterol regulatory element-binding protein-2 (SREBP-2), and low-density lipoprotein receptors (LDLR), had intense interaction [36].

In our study, the hypolipidemic effect of *D. odorifera* leaves was exhibited in high-fat diet-induced hyperlipidemic rats. The relationship between the components and the underlying mechanisms in the hypolipidemic effect was investigated using network pharmacology-based approaches. Finally, the predicted targets were verified by western blotting.

## 2. Material and Methods

### 2.1. Material and Chemicals

The leaves of *D. odorifera* were collected from the Jianfengling area, Sanya City, Hainan Province, China, and identified by an associate researcher, Zhou Hong, Institute of Tropical Botany, Chinese Academy of Forestry. A voucher specimen (No. 20200123) was deposited in the Key Laboratory of Modern Preparation of Traditional Chinese Medicine, Ministry of Education, Jiangxi University of Chinese Medicine, China.

The TC assay kit (Lot No. 2020021001), TG assay kit (Lot No. 2020051901), HDL-C assay kit (Lot No. 2020041001), and LDL-C assay kit (Lot No. 2020061102) were offered by Epnkan Biological Technology Co., China. 3-Hydroxy-3-methylglutaryl-coenzyme A reductase (HMGCR) (Lot No. EG20200902) was purchased from EnoGene Biological Technology Co., China. The HMGCR ELISA kit (Lot No. 20210804.60597R), interleukin-1*β* (IL-1*β*) ELISA kit (Lot No. 20210804.60013R), interleukin-6 (IL-6) ELISA kit (Lot No. 20210804.60023R), interleukin-18 (IL-18) ELISA kit (Lot No. 20210804.60033R), and tumor necrosis factor-alpha (TNF-*α*) ELISA kit (Lot No. 20210804.60080R) were obtained from Beijing RIGORBIO Technology Development Co., Ltd., China. The total superoxide dismutase (SOD) assay kit (Lot No. 20210802), catalase (CAT) assay kit (Lot No. 20210805), and glutathione peroxidase (GSH-Px) assay kit (Lot No. 20210803) were purchased from Nanjing Jiancheng Bioengineering Institute, China. Antibodies, including HMGCR (BSM-52822R), LDLR (bs-0705R), and sterol regulatory element-binding protein cleavage-activating protein (SCAP) (bs-3862R), were purchased from Bioss, China. The SREBP-2 antibody (DF7601) was purchased from Internal Affinity Biosciences, USA. The *β*-actin (4D3) polyclonal antibody (AP6007M) was obtained from Bioworld Technology, Inc, USA. Distilled water was prepared by the laboratory. All other chemicals were of analytical grade.

### 2.2. Extract Preparation

*D. odorifera* leaves (10 kg) were combined with 10 times the volume of 70% ethanol under reflux twice successively for each 2 h, followed by filtration. The combined extracts were then rotary evaporated at 65°C and lyophilized. The ethanol extract (1.5 kg) was stored at 4°C. The polyphenol content in the ethanol extract was 12.19 mg gallic acid equivalent g^-l^ dry weight using the Folin–Ciocalteu method [4].

### 2.3. Animals

Sprague Dawley male rats (200 ± 20 g, age 7-8 weeks) were supplied by Hunan SJA Laboratory Animal Co., Ltd (Hunan, China). The rats were kept at room temperature (24–26°C, 65% ± 10% humidity, and a 12/12 h light/dark cycle) with commercial rat normal standard chow (Hunan SJA Laboratory Animal Co., Ltd., Hunan, China) and water *ad libitum*.

After allowing a week for adaptation, all rats were randomly assigned into six groups (*n* = 10). The rats in the normal group were fed a standard basal diet, whereas rats in the other 5 groups were fed a high-fat diet (52.6% regular diet, 20.0% sucrose, 15.0% lard, 1.2% cholesterol, 0.2% bile salts, 10% casein, 0.6% calcium hydrophosphate, and 0.4% mountain flour) to obtain the hyperlipidemic model [37]. After 2 weeks, the rats in the normal and model groups were intragastrically administered 10 ml·kg^−1^ body weight (BW) of distilled water once a day. The rats in group 3 were intragastrically administered 7 mg·kg^−1^·BW of atorvastatin, and those in groups 4–6 rats were intragastrically administered the extract at a dose of 0.4, 0.8, and 1.6 g·kg^−1^·BW once a day, respectively. The dose fixation of the extract was referenced in the previous study [38]. After four weeks, the rats were fasted for 12 h and anesthetized by intraperitoneal injection of a 20% urethane solution (1 ml·100 g^−1^ BW). Blood was collected from the orbit, left at room temperature for 15 min, and then centrifuged at 3500 rpm (4°C, 10 min). The serum obtained was stored at −80°C until biochemical analysis. The livers were dissected, washed with saline, and weighed. Part of the liver was fixed with 10% formalin for pathological histology, and the remaining portion was stored at −80°C until analysis.

### 2.4. Serum Lipids

The serum lipid (TC, TG, LDL-C, and HDL-C) levels were determined using commercial assay kits from Epnkan Biological Technology Co., China.

### 2.5. HMGCR Activity

The levels of HMGCR in the plasma were measured using a Thermo Multiskan MK3 microplate reader (Finland).

### 2.6. Hematoxylin and Eosin (H&E) Staining

Liver tissue specimens were fixed with 10% formalin for 72 h at room temperature. After fixation, sections perpendicular to the anterior-posterior axis of the tissue were dehydrated with graded ethanol, cleared with xylene, and embedded in paraffin. The sections (4 *µ*m thick) were mounted on glass slides, rehydrated with distilled water, and stained with H&E. The state of the tissue was observed under a light microscope [37].

### 2.7. Antioxidant Enzyme Activities

The antioxidant enzyme (SOD, CAT, and GSH-Px) activities in the plasma were determined using commercial assay kits with a Beckman Coulter UniCel DxC 600 Synchron automatic biochemical analyzer (USA).

### 2.8. Anti-inflammatory Activities

The levels of IL-1*β*, IL-6, IL-18, and TNF-*α* in the plasma were measured using a Thermo Multiskan MK3 microplate reader (Finland).

### 2.9. Network Pharmacological Analysis

#### 2.9.1. Screening of Candidate Biotargets and Network Construction

The main components from *D. odorifera* leaves were retrieved from the literature databases. The web-available database of the Swiss Target Prediction (http://www.swisstargetprediction.ch/) was used to predict their candidate targets. Furthermore, the targets related to hyperlipidemia were mined in Online Mendelian Inheritance in Man database (https://www.omim.org/), and the search keywords were standardized as the MESH keywords “hyperlipidemia,” “hyperlipemia,” “high-fat blood disease,” and “high-fat blood” [39]. The intersection of the predicted targets and disease targets was carried out via Venny 2.1.0. The intersectional targets with a degree of ≥5 were normalized to the genetic names in the PubMed gene profile for Homo sapiens. Intersectional targets with an interaction degree of ≥10 with *D. odorifera* leaves were visualized using Cytoscape software 3.7.2.

#### 2.9.2. Biological Function and Pathway Enrichment Analyses of *D. odorifera f* Extract-Treating Hyperlipidemia

Gene ontology (GO) analysis and KEGG signaling pathway analysis were performed with David 6.8 (https://david.ncifcrf.gov/), with official-gene-symbol, threshold *P* < 0.05, screening condition *P* < 0.05, gene list, and Homo sapiens for differentially expressed genes [39]. GO consists of three aspects: biological process, cellular component, and molecular function. Differentially expressed genes were mapped to the KEGG signaling pathways with *P* < 0.05 and a false discovery rate (FDR) < 0.05. Data were imported into the online drawing Image GP platform (http://www.ehbio.com/ImageGP/) to output visualization figures.

### 2.10. Western Blotting

Total proteins were obtained from rat hepatic tissues, which were homogenized in RIPA buffer supplemented with phenylmethylsulfonyl fluoride and a protease inhibitor cocktail. Protein samples were separated on 10% separation gels and then transferred onto polyvinylidene fluoride membranes. After blocking with 5% fetal bovine serum for 1 h, the membranes were incubated separately with the primary rabbit polyclonal antibodies against HMGCR (1 : 1500), LDLR (1 : 1500), SREBP-2 (1 : 2000), SCAP (1 : 1500), and the mouse polyclonal antibodies against *β*-actin(1 : 10000) overnight at 4°C. After washing, the membranes were incubated with appropriate secondary antibodies at room temperature for 45 minutes [37]. Finally, the membranes were treated according to the protocol for the enhanced chemiluminescence detection kit, and protein bands were observed using Tanon 4200.

### 2.11. Statistical Analysis

All data are presented as the mean ± standard deviation. Statistical analysis was performed using one-way analysis of variance and Student's *t*-test with GraphPad Prism software (version 8.3 for Windows, La Jolla, CA, USA). *P* < 0.05 was considered the criterion of significance.

## 3. Results

### 3.1. Hypolipidemic Effects of *D. odorifera* Leaf Extract

**Figure 1:**
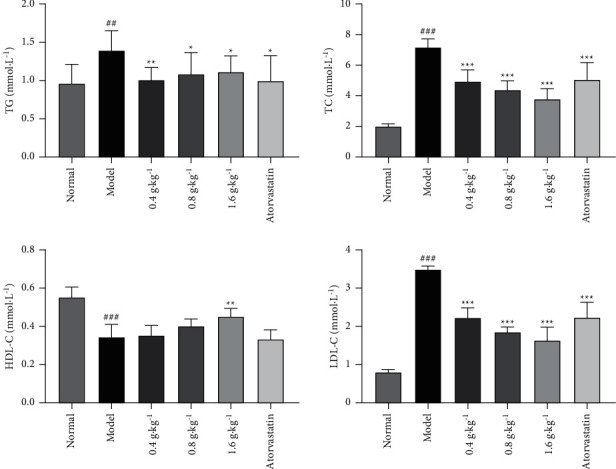
Effects of *D. odorifera* leaf extract on TG, TC, HDL-C, and LDL-C in rats fed on high-fat diet or normal diet. Data are expressed as mean ± standard deviation, *n* = 10. ^#^*P* < 0.05, ^##^*P* < 0.01, and ^###^*P* < 0.001 compared with the normal group. ^*∗*^*P* < 0.05, ^*∗∗*^*P* < 0.01, and ^*∗∗∗*^*P* < 0.001 compared with the model group.

Compared with the levels in the normal group, the levels of serum TG, TC, and LDL-C in the model group were significantly higher (*P* < 0.01 and *P* < 0.001), whereas the serum level of HDL-C in the model group was significantly lower (*P* < 0.001). These findings demonstrated that the hyperlipidemic rat model was successfully established. Furthermore, the TG, TC, and LDL-C levels in all the groups treated with the extract were significantly lower than those in the model group (*P* < 0.05, *P* < 0.01, and *P* < 0.001). In addition, the HDL-C level in the extract (1.6 g·kg^−1^)-treated group was significantly higher (*P* < 0.01) than that in the model group, but that in 0.4, 0.8 g·kg^−1^-treated groups was not significantly different, as shown in Figure 1. These results showed that the extract from *D. odorifera* leaves exhibited the effect of alleviating disorders of lipoprotein metabolism.

### 3.2. HMGCR Activity

**Figure 2:**
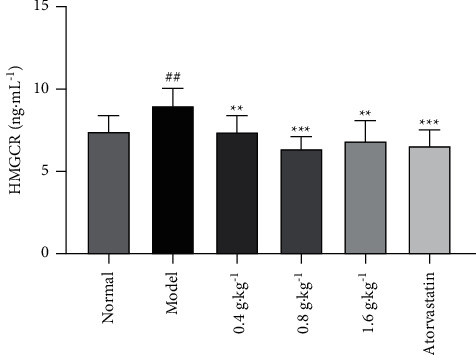
Effects of *D. odorifera* leaf extract on HMGCR in rats fed on high-fat diet or normal diet. Data are expressed as mean ± standard deviation, *n* = 10. ^#^*P* < 0.05, ^##^*P* < 0.01, and ^###^*P* < 0.001 compared with the normal group. ^*∗*^*P* < 0.05, ^*∗∗*^*P* < 0.01, and ^*∗∗∗*^*P* < 0.001 compared with the model group.

HMGCR is the key enzyme in cholesterol biosynthesis. As shown in Figure 2, compared with that in the normal group, the HMGCR level in the plasma of the model group was markedly increased (*P* < 0.01) in rats fed a high-fat diet for 6 weeks. Compared with the model group, all the groups treated with the extract showed significantly lower levels of HMGCR (*P* < 0.01 and *P* < 0.001). We suggested HMGCR is probably a key factor in its hypolipidemic mechanism.

### 3.3. H&E Staining

**Figure 3:**
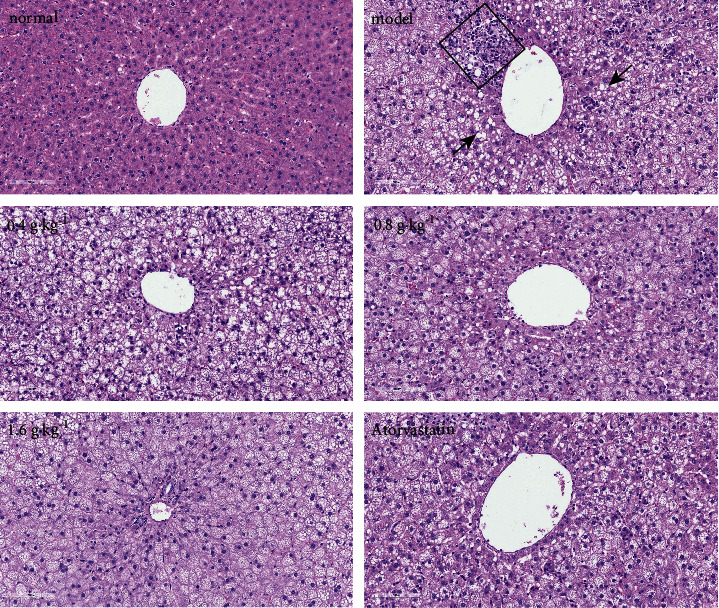
H&E staining of liver tissue (200×). The black rectangle represents inflammatory cell infiltration, and the black arrow represents the lipid vacuole.

The H&E staining results indicated that the hepatocytes appeared regular and that the structure of hepatic lobules was clear in the normal group. A large number of lipid vacuoles appeared in the hepatocytes of the model group. Cell nuclei were squeezed and shifted to one side by large lipid droplets, along with obvious steatosis. Moreover, extensive inflammatory cell infiltration occurred in lobules. After treatment with the extract, the degree of steatosis and inflammatory cell infiltration were markedly attenuated compared with those in the model group, as shown in Figure 3.

### 3.4. Antioxidant Effect of *D. odorifera* Leaves *In Vivo*

**Figure 4:**
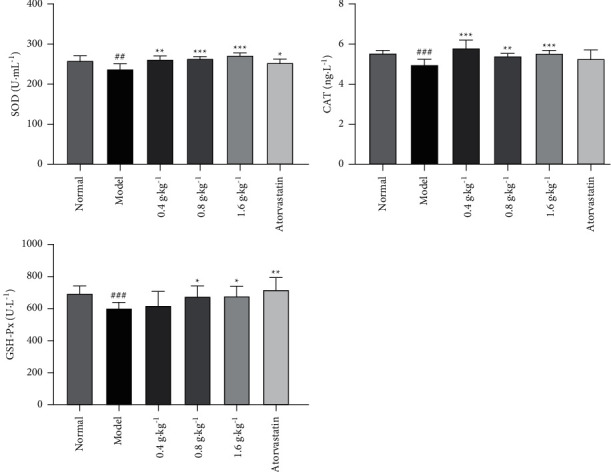
Effects of *D. odorifera* leaf extract on SOD, CAT, and GSH-Px levels in rats fed on high-fat diet or normal diet. Data are expressed as mean ± standard deviation, *n* = 10. ^#^*P* < 0.05, ^##^*P* < 0.01, and ^###^*P* < 0.001 compared with the normal group. ^*∗*^*P* < 0.05, ^*∗∗*^*P* < 0.01, and ^*∗∗∗*^*P* < 0.001 compared with the model group.

In this study, compared with the normal group, hyperlipidemic rats exhibited decreased plasma SOD, CAT, and GSH-Px activities, as shown in Figure 4. *D. odorifera* leaf extract at three different doses markedly increased the lowered SOD and CAT activities (*P* < 0.01 and *P* < 0.001) in the plasma of hyperlipidemic rats. The reduced GSH-Px activity was increased in the extract (0.8 and 1.6 g·kg^−1^)-treated groups (*P* < 0.05). These results indicated that the ethanol extract of *D. odorifera* leaves exhibited a significant antioxidant effect in hyperlipidemic rats.

### 3.5. Anti-Inflammatory Effect of *D. odorifera* Leaf Extract *In Vivo*

**Figure 5:**
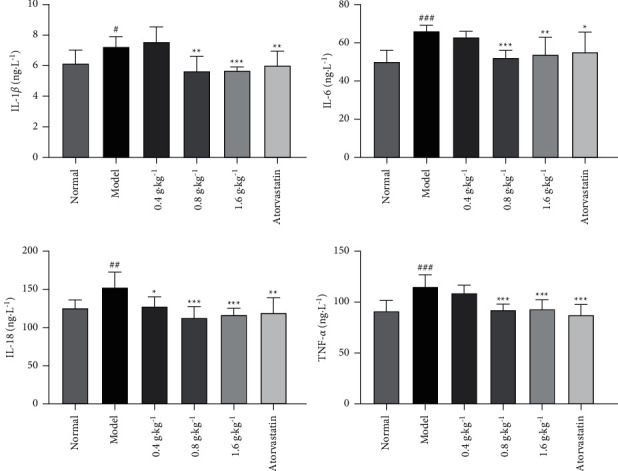
Effects of *D. odorifera* leaf extract on IL-1*β*, IL-6, IL-18, and TNF-*α* levels in rats fed on high-fat diet or normal diet. Data are expressed as mean ± standard deviation, *n* = 10. ^#^*P* < 0.05, ^##^*P* < 0.01, and ^###^*P* < 0.001 compared with the normal group. ^*∗*^*P* < 0.05, ^*∗∗*^*P* < 0.01, and ^*∗∗∗*^*P* < 0.001 compared with the model group.

The anti-inflammatory activity of the extract was detected *in vivo*. Compared with levels in the normal group, the levels of IL-1*β*, IL-6, IL-18, and TNF-*α* in the plasma of the model group were markedly elevated in rats fed a high-fat diet for 6 weeks. Compared with the model group, the extract-treated groups showed significantly lower levels of IL-18 (*P* < 0.05 and *P* < 0.001). Simultaneously, the levels of IL-1*β*, IL-6, and TNF-*α* in the hyperlipidemic rats were dramatically attenuated with the treatment at the levels of 0.8 and 1.6 g·kg^−1^ (*P* < 0.01 and *P* < 0.001), whereas those in the extract (0.4 g·kg^−1^)-treated group were not significantly changed, as shown in Figure 5.

### 3.6. Network Pharmacological Analysis

#### 3.6.1. Screening of Candidate Biotargets and Network Construction

**Figure 6:**
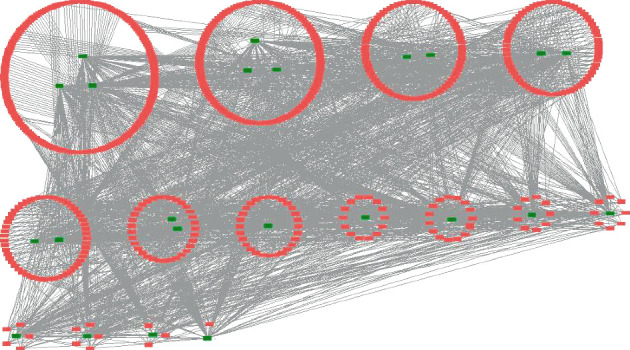
The compounds-targets network of *D. odorifera* leaves against hyperlipidemia (green represents the ingredients, and red represents the targets).

Twenty-four components extracted from *D. odorifera* leaves were found in literature databases, including 9 fatty acids (lauric acid, myristic acid, tetradecenoic acid, palmitic acid, palmitoleic acid, oleic acid, linoleic acid, linolenic acid, and eicosanoic acid), 6 flavonoids (biochanin A, 6-hydroxy-biochanin A, tectorigenin, genistein, prunetin, and isoliquiritigenin), 3 aryl glycosides (icariside F2, benzyl alcohol *β*-vicianoside, and phenethyl alcohol *β*-vicianoside), 2 megastigmane glucosides (icariside B1 and icariside B6), 1 monoterpene [(3S)-6,7-dihydroxy-6,7-dihydrolinalool], 1 alkyl glucoside (butane-2,3-diol 2-O-*β*-D-glucoside), 1 purine (adenine), and 1 amino acid (gamma-aminobutyric acid). However, no targets were retrieved for (3S)-6,7-dihydroxy-6,7-dihydrolinalool from the Swiss Target Prediction database. Thus, 35 targets with a degree of ≥10 were pooled for 23 components from *D. odorifera* leaves. The target gene names and the compounds-target network for the treatment of hyperlipidemia with *D. odorifera* leaf extract, were shown in Table 1 and Figure 6, respectively.

#### 3.6.2. Biological Functions and Pathway Enrichment Analysis of *D. odorifera* Leaf Components in the Treatment of Hyperlipidemia

**Figure 7:**
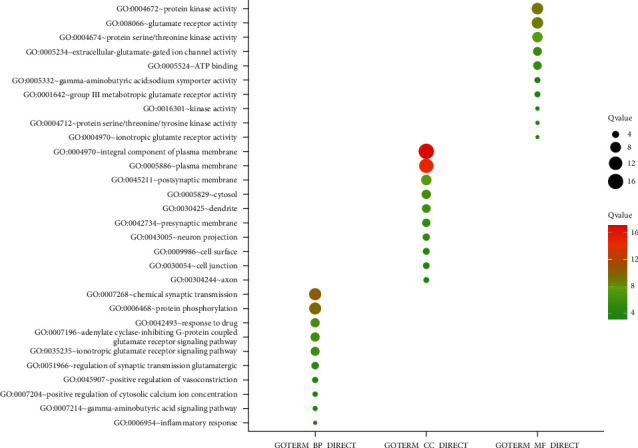
GO function enrichment results of *D. odorifera* leaves in the treatment of hyperlipidemia.

GO analysis revealed biological processes including chemical synaptic transmission, protein phosphorylation, and adenylate cyclase-inhibiting and G-protein-coupled glutamate receptor signaling pathway. Enrichment of cellular components indicated that integral components of the plasma membrane, postsynaptic membrane, and cytosol were involved. Protein kinase activity, glutamate receptor activity, and protein serine/threonine kinase activity participated in the molecular function of *D. odorifera* leaf components and antihyperlipidemic targets, as shown in Figure 7.

**Figure 8:**
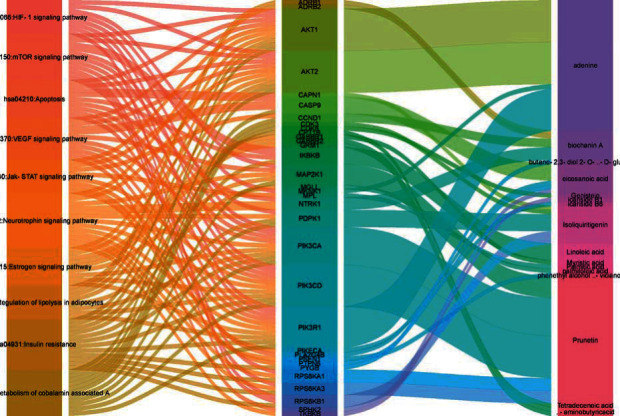
KEGG enrichment of *D. odorifera* leaves in the treatment of hyperlipidemia.

The KEGG signaling pathway enrichment results were plotted according to the -log (FDR) value. Moreover, the top 10 signaling pathways were associated with the targets and components in the Sankey diagram, as shown in Figure 8. The thickness of the connecting line is expressed by the -log (FDR) value. As a result, the top 10 signaling pathways of *D. odorifera* leaf extract involved in the treatment of hyperlipidemia were the metabolism of cobalamin-associated A, HIF-1 signaling pathway, insulin resistance, regulation of lipolysis in adipocytes, mTOR signaling pathway, apoptosis, VEGF signaling pathway, estrogen signaling pathway, neurotrophin signaling pathway, and Jak-STAT signaling pathway.

### 3.7. Western Blotting

**Figure 9:**
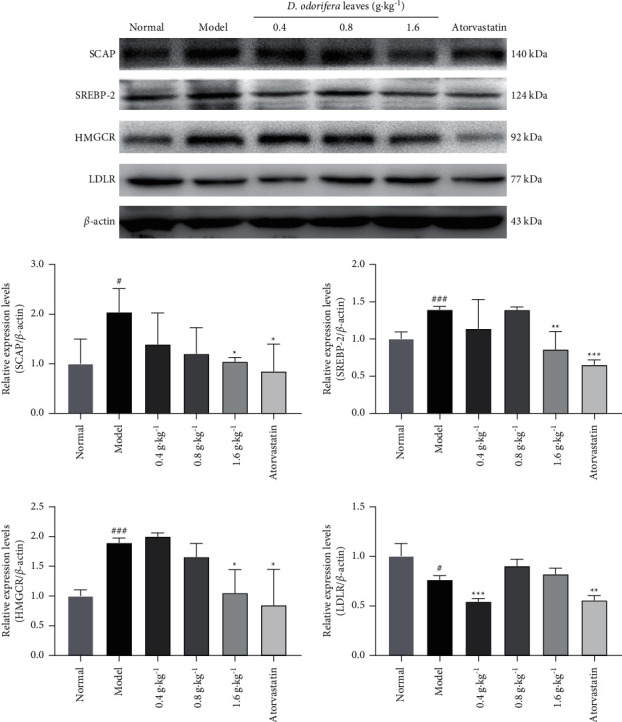
Western blotting. Effect of *D. odorifera* leaves on the protein levels of SCAP, SREBP-2, HMGCR, and LDLR in the liver.

The expression of SREBP-2, SCAP and HMGCR in liver tissues was dramatically higher, whereas that of LDLR was dramatically lower in the model group than in the normal group after feeding a high-fat diet for six weeks, as shown in Figure 9. Compared with those in the model group, the expression levels of the SREBP-2, SCAP, and HMGCR proteins in the liver tissue of hyperlipidemic rats were significantly downregulated after treatment with the extract (1.6 g·kg^−1^) for four weeks. Although it did not reach the level of significance, there was a clear increasing trend in that the expression level of the LDLR protein, which was increased after four weeks of treatment with the extract (0.8 and 1.6 g·kg^−1^).

## 4. Discussion

Hyperlipidemia is caused by excessive lipid intake, lipid synthesis, and metabolic disorders [36]. It is accompanied by the elevation of serum TC, TG, and LDL-C levels and a reduction in HDL-C levels. Moreover, HMGCR is the rate-limiting enzyme in cholesterol biosynthesis and plays an important role in cholesterol biosynthesis [40]. In this study, the extract of *D. odorifera* leaves ameliorated dyslipidemia, with a reduction in blood lipid (TG, TC, and LDL-C) concentrations and an elevation in HDL-C levels in the serum. The extract also suppressed the HMGCR activity in the plasma and its protein expression in the liver of hyperlipidemic rats. In addition, the imbalance of lipid levels between lipid synthesis and lipid consumption in the liver induces excessive lipid accumulation in the liver [41, 42]. *D. odorifera* leaf extract inhibited hepatocyte steatosis and inflammatory cell infiltration in the liver. Moreover, the extract suppressed HMGCR protein expression in the livers of hyperlipidemic rats. Our results suggested that the extract inhibited fat accumulation and steatosis in hepatocytes, thus alleviating liver injury. Flavonoids such as genistein, biochanin A, tectorigenin, and prunetin are abundant in *D. odorifera* leaves [25]. Additionally, genistein and biochanin A are the main components of *D. odorifera* leaves [27]. In the previous reports, genistein had a hypolipidemic effect on menopausal women with hyperlipidemia [43] and ameliorated dyslipidemia and hepatic steatosis in hyperlipidemic hmsters [32] and diabetic mice [44]. Biochanin A mitigates dyslipidemia in hyperlipidemic mice [33], atherosclerotic mice [45], and diabetic rats [46]. In addition, tectorigenin and prunetin improved the effect of improving dyslipidemia in diabetic rats and obese mice, respectively [30, 31]. Therefore, the hypolipidemic effect of *D. odorifera* leaf extract might be attributed to the presence of flavonoids.

Oxidative stress is closely related to hyperlipidemia-related tissue impairment [47, 48]. SOD, CAT, and GSH-Px, as the main factors in the antioxidant enzyme system, work together to reduce the generation of active oxygen radicals, inhibit lipid peroxidation and prevent products of metabolization from undermining bodies, and maintain the balance in reactive oxygen species [49]. According to a previous report, the extract of *D. odorifera* leaves exhibits a significant scavenging effect on free radicals *in vitro* [22]. In this study, after the treatment with the extract, the levels of SOD, CAT, and GSH-Px in plasma were increased in hyperlipidemic rats. In the previous report, biochanin A elevated SOD levels and suppressed malondialdehyde (MDA) levels in the serum. It also reduces MDA levels in liver tissues of the high-fat diet-induced hyperlipidemic mice [33]. Genistein also exhibits the ability to increase SOD levels and decrease MDA levels in hyperlipidemic hamsters [32]. Thus, it is probable that flavonoids in the extract relieved the oxidative stress caused by hyperlipidemia.

Inflammation also plays a crucial role in the initiation and progression of hyperlipidemia. It is regulated by proinflammatory mediators and cytokines, including IL-1*β*, IL-6, IL-18, and TNF-*α* [50]. In our study, *D. odorifera* leaf extract had an anti-inflammatory effect in hyperlipidemic rats. Following treatment with the extract, the plasma levels of proinflammatory cytokines were obviously suppressed in hyperlipidemic rats. Fatty acids exert their inflammatory effects via the activation of the nuclear factor-kappa B signaling pathway, leading to the secretion of these proinflammatory cytokines [51]. Flavonoids are closely related to anti-inflammatory activity [52]. Both genistein and biochanin A significantly reduced the levels of free fatty acids in hyperlipidemic hamsters and obese rats [32, 53]. Quercetin, genistein, and naringenin alleviated TNF-*α* and IL-6 levels in rats with high-fructose and high-fat diet-induced metabolic syndrome rats, respectively [54]. Therefore, we suggested that the hypolipidemic effect of *D. odorifera* leaf extract was achieved by reducing lipid accumulation, thus alleviating inflammation in hyperlipidemic rats.

Regarding the network pharmacology results, the highest-scoring signaling pathway that was enriched in the KEGG signaling pathway analysis was the metabolism of cobalamin associated A pathway. The metabolism of cobalamin associated A is a nominally generalized metabolic signaling pathway. This signaling pathway is usually involved in clinical research on nutrient metabolism, especially in special populations, such as children, pregnant women, or aged people [55–57]. The only conserved genes that could be found in the metabolism of cobalamin associated A pathway were CASP9, MAP2K1, CDK6, CCND1, PIK3CA, PDPK1, CDK3, AKT2, PIK3CD, AKT1, and PIK3R1; however, the SCAP, SREBP-2, HMGCR, and LDLR genes had a higher degree in the network and were considered the novel targets that are complementary to the signaling pathway.

Disorders of lipid metabolism are closely related to hyperlipidemia. SREBP-2, SCAP, HMGCR, and LDLR are key targets for the cholesterol biosynthesis [58]. SREBPs are major transcription factors that activate the expression of genes involved in lipid synthesis. SREBP-2, as one of the three SREBP isoforms, is relatively specific to the regulation of cholesterol synthesis and uptake [59]. SCAP, which acts as an escort chaperone for SREBP-2, induces the transcription of genes involved in the cholesterol synthesis and uptake by transporting the membrane-bound transcription factor called SREBP-2 from the ER to the Golgi apparatus for proteolytic activation [60, 61]. HMGCR is a crucial enzyme in cholesterol synthesis [58]. LDLR is a cell surface receptor that regulates cholesterol uptake by mediating the endocytosis of cholesterol-rich LDLs [62]. The SCAP/SREBP/INSIG1 trio promotes hepatic lipid remodeling to protect the liver from lipotoxic insults associated with the progression of nonalcoholic steatohepatitis progression [63]. The downregulation of SREBP-2 and HMGCR protein expression might attenuate high-fat diet-induced hyperlipidemia [64, 65]. In this study, the downregulation of SCAP, SREBP-2, and HMGCR expression, and the upregulation of LDLR expression subsequently attenuated lipid accumulation. These results are similar to those in previous reports that examined some flavonoids from the extract [66] and suggest that the mechanism by which *D. odorifera* leaf extract ameliorates hyperlipidemia is related to the downregulation of target proteins, such as HMGCR, SCAP, and SREBP-2, and the upregulation of LDLR.

## 5. Conclusion

In summary, *D. odorifera* leaf extract ameliorated the symptoms of hyperlipidemia and its related oxidative stress and inflammatory response in high-fat diet-induced hyperlipidemic rats. The hypolipidemic effect may be mediated through downregulating targets related to cholesterol biosynthesis, such as SCAP, SREBP-2, and HMGCR, and upregulating LDLR, resulting in the alleviation of hyperlipidemia. These findings increased the accumulating evidence on the hypolipidemic effect of *D. odorifera* leaf extract.

## Figures and Tables

**Table 1:** Targets gene name with degree ≥10.

No.	Gene name	Degree
1	SCAP	18
2	SREBP2	18
3	HMGCR	18
4	LDLR	18
5	CYP19A1	17
6	MMP2	14
7	MMP9	14
8	PPARA	14
9	AKR1B1	13
10	EGFR	13
11	ESR1	13
12	ESR2	13
13	MMP13	13
14	ADORA2A	12
15	AKR1C3	12
16	CDC25A	12
17	MMP12	12
18	PTPN1	12
19	ADORA1	11
20	ALOX12	11
21	CDC25B	11
22	FNTA, FNTB	11
23	MCL1	11
24	MME	11
25	MMP14	11
26	MMP3	11
27	ACE	10
28	ADORA3	10
29	ALOX15	10
30	CA12	10
31	CA2	10
32	IGFBP3	10
33	KDM2A	10
34	MMP8	10
35	TYR	10

## Data Availability

The data used to support this study are available from the corresponding author upon request.
